# Inhibition of endoplasmic reticulum glucosidases is required for *in vitro* and *in vivo* dengue antiviral activity by the iminosugar UV-4

**DOI:** 10.1016/j.antiviral.2016.03.001

**Published:** 2016-05

**Authors:** Kelly L. Warfield, Emily M. Plummer, Andrew C. Sayce, Dominic S. Alonzi, William Tang, Beatrice E. Tyrrell, Michelle L. Hill, Alessandro T. Caputo, Sarah S. Killingbeck, P. Robert Beatty, Eva Harris, Ren Iwaki, Kyoko Kinami, Daisuke Ide, J.L. Kiappes, Atsushi Kato, Michael D. Buck, Kevin King, William Eddy, Mansoora Khaliq, Aruna Sampath, Anthony M. Treston, Raymond A. Dwek, Sven G. Enterlein, Joanna L. Miller, Nicole Zitzmann, Urban Ramstedt, Sujan Shresta

**Affiliations:** a Emergent Virology LLC, Gaithersburg, MD 20879, USA; b La Jolla Institute for Allergy and Immunology, La Jolla, CA 92037, USA; c Oxford Glycobiology Institute, Department of Biochemistry, University of Oxford, Oxford, United Kingdom; d Division of Infectious Diseases and Vaccinology, School of Public Health, University of California-Berkeley, Berkeley, CA, USA; e Department of Hospital Pharmacy, University of Toyama, Toyama, Japan; f Integrated Biotherapeutics, Gaithersburg, MD 20878, USA; g Unither Virology LLC, Silver Spring, MD 20910, USA

**Keywords:** Iminosugar, UV-4B, Dengue, Antibody-dependent enhancement, Antiviral, Glucosidase

## Abstract

The antiviral activity of UV-4 was previously demonstrated against dengue virus serotype 2 (DENV2) in multiple mouse models. Herein, step-wise minimal effective dose and therapeutic window of efficacy studies of UV-4B (UV-4 hydrochloride salt) were conducted in an antibody-dependent enhancement (ADE) mouse model of severe DENV2 infection in AG129 mice lacking types I and II interferon receptors. Significant survival benefit was demonstrated with 10–20 mg/kg of UV-4B administered thrice daily (TID) for seven days with initiation of treatment up to 48 h after infection. UV-4B also reduced infectious virus production in *in vitro* antiviral activity assays against all four DENV serotypes, including clinical isolates. A set of purified enzyme, *in vitro*, and *in vivo* studies demonstrated that inhibition of endoplasmic reticulum (ER) α-glucosidases and not the glycosphingolipid pathway appears to be responsible for the antiviral activity of UV-4B against DENV. Along with a comprehensive safety package, these and previously published data provided support for an Investigational New Drug (IND) filing and Phases 1 and 2 clinical trials for UV-4B with an indication of acute dengue disease.

## Highlights

• The iminosugar UV-4B has *in vitro* activity against all 4 dengue virus serotypes.
• Inhibition of ER α-glucosidases is responsible for UV-4B activity against dengue.
• *In vivo* efficacy studies inform clinical trial design for UV-4B treatment of dengue.

## 1 Main text

Dengue virus (DENV) can cause a range of disease manifestations from asymptomatic infection to severe dengue disease, which can result in death (Halstead, 2007). DENV is estimated to infect up to 390 million people worldwide annually (Bhatt et al., 2013). The resultant disease is a global health burden that strains medical systems in tropical and subtropical regions, where the virus circulates in *Aedes* mosquito populations. Currently no approved vaccine or antiviral therapy for DENV exists (Coller et al., 2010, Gubler, 1998, Julander et al., 2011). Four distinct serotypes of DENV (designated DENV1-4) infect humans, and epidemiological studies indicate that severe disease occurs most often during secondary infection with a heterologous serotype. A leading hypothesis to explain this phenomenon, the antibody-dependent enhancement (ADE) hypothesis, states that the presence of cross-reactive, non-neutralizing antibodies generated during primary infection or acquired passively at birth contributes to severe disease upon infection by another serotype (Halstead, 2007).

Iminosugars have been explored as antiviral agents against enveloped viruses because they demonstrate selective inhibition of viral assembly and secretion, presumably through the inhibition of the host endoplasmic reticulum (ER)-resident glycosylation pathway, leading to misfolding of viral glycoproteins (Chang et al., 2013, Durantel et al., 2005, Dwek et al., 2002, Mehta et al., 1998). An antiviral agent that targets a host pathway could avoid challenges associated with directly acting antivirals, including viral susceptibility, virus heterogeneity and the rapid emergence of drug-resistant mutants (Sayce et al., 2010). We recently conducted a study of DENV evolution under pressure with the host-targeted iminosugar UV-4B, the hydrochloride salt of UV-4 (*N*-(9′-methoxynonyl)-1-deoxynojirimycin or M*O*N-DNJ). This study demonstrated a high genetic barrier to escape mutations, supporting the theory that host-targeted therapies should show significantly reduced likelihood for development of resistance-conferring mutations (Plummer et al., 2015). We have previously characterized the efficacy of UV-4 and UV-4B *in vivo* in mouse models of severe dengue disease via both direct infection (virus only) and ADE (virus plus exogenous DENV-specific antibodies) studies. UV-4 protected mice from lethal DENV infection in a dose-dependent manner, reduced viral titer in tissues, and decreased cytokine levels in circulation (Perry et al., 2013). We also showed that initiation of UV-4 treatment could be delayed until 48 h after infection when a high dose was administered [100 mg/kg given thrice daily (TID)]. Importantly, administration of UV-4 did not alter antibody responses after DENV infection. Together, these findings supported further investigation of UV-4B (the hydrochloride salt was selected for development).

Previously, the *in vitro* activity of UV-4 was described against DENV2 (Perry et al., 2013). In the current study, the antiviral activity of UV-4 against DENV1-4 was assessed using an infectious virus yield-reduction assay similar to previous reports (Warfield et al., 2015). Briefly, UV-4B was tested for activity at 6–8 concentrations (two-fold dilutions starting at 125–500 μM, each in duplicate) and the collected supernatants were quantitated for functional DENV using an immunoplaque assay. As shown in Table 1, UV-4B inhibited all the DENV isolates tested *in vitro*. The 50% inhibitory concentration (IC_50_) values for UV-4B in these studies ranged from 2.10 μM (DENV1 SH29177) to 86.49 μM (DENV3 H87). The selectivity index, calculated from the IC_50_ and CC_50_ (50% cytotoxic concentration) of UV-4 in Vero cells (CC_50_ > 1 mM), ranged from >11.6 to >477. Using a similar assay set up in BHK cells, the IC_50_ of UV-4B against the mouse challenge virus DENV-2 S221 was found to be 38.98 μM.

**Fig. 1:**
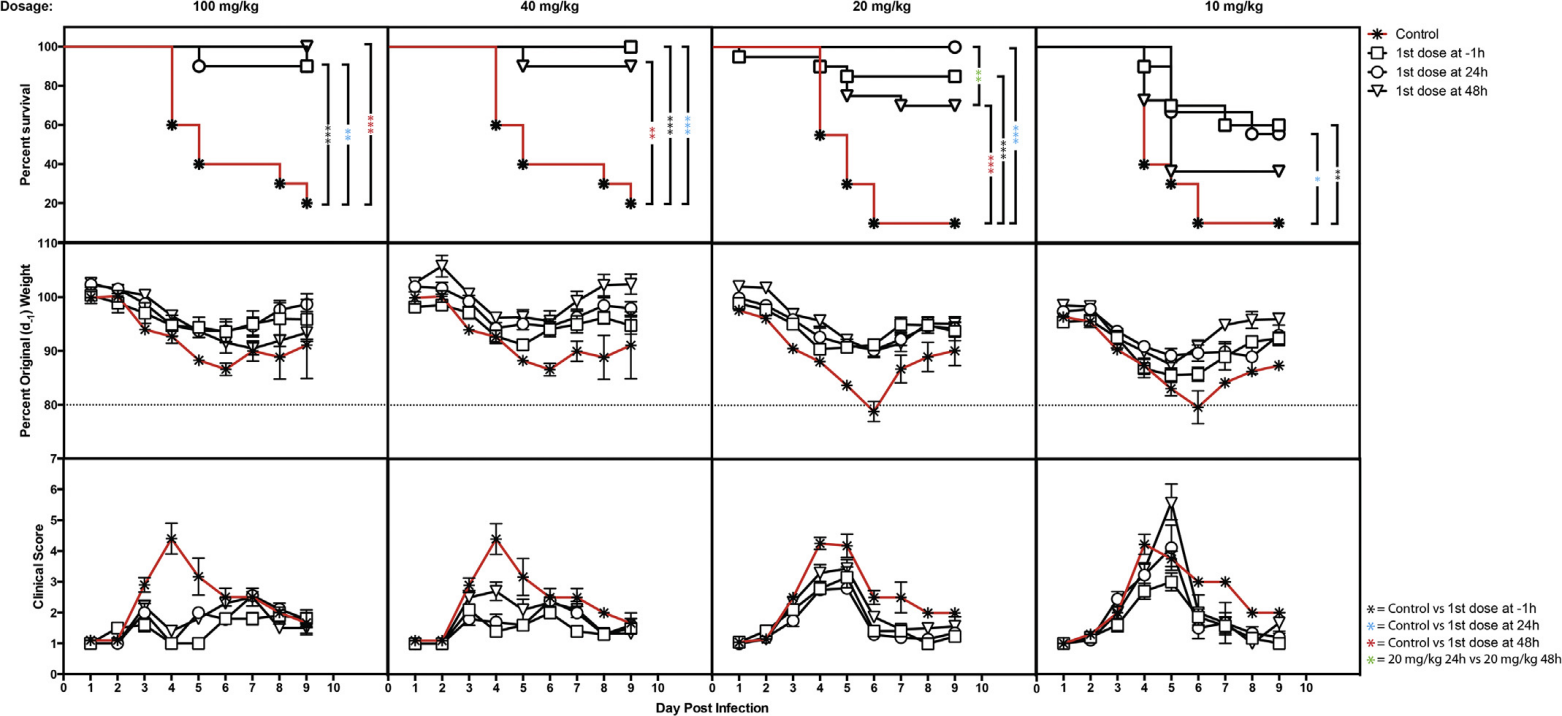
**Therapeutic window of various dose levels of UV-4B in lethal dengue ADE mouse model**. Groups of AG129 mice (n = 10) received the first treatment dose of 100, 40, 20 or 10 mg/kg of UV-4 (dosed orally as UV-4B) or vehicle 1 h before or 24 or 48 h after infection with DENV2 S221 in the presence of DENV-specific antibody clone 2H2 at a dose of ∼1 LD_90_ (10^9^ genomic equivalents); treatment continued every 8 h daily for seven days once initiated. (Top row) Survival data are plotted as percent survival against days post-infection. Asterisks denote statistical significance as determined by the Gehan–Breslow–Wilcoxon test (*, p = 0.05; **, p = 0.01; ***, P < 0.001). (Middle row) The mean percent weights for each group are plotted compared to their percent weight on Day -1 (baseline) against days post-infection. Error bars represent the standard error mean (SEM). (Bottom row) The mean health score, based on a standardized system with values from 1 to 7 given daily to each mouse, with the SEM for each group plotted against days post-infection.

In the *in vivo* studies described here, mice were dosed orally with varying concentrations (100, 40, 20 or 10 mg/kg) of UV-4 TID as aqueous UV-4B solution, starting 1 h before or 24 or 48 h after lethal ADE DENV2 challenge (Plummer and Shresta, 2014, Tang et al., 2015, Zellweger and Shresta, 2014), and every 8 h thereafter for a total of seven days of treatment. Weight loss and health were monitored daily throughout infection and dosing, and continued for three days following the dosing period to quantitate changes in disease course. Health was determined on the basis of clinical scores ranging from 1 (completely healthy) to 7 (dead), based on a detailed rubric that includes evaluation of hunched posture, ruffling of fur, inset of eyes, and lethargy as previously described (Stavale et al., 2015). Based on extensive studies of this mouse model, animals that lost >20% of their original weight or had a clinical score ≥ 5 were considered to have succumbed to dengue disease and were euthanized immediately. Mice dosed with 100 mg/kg TID starting at −1, +24, or +48 h relative to infection exhibited survival rates of 90, 90, and 100%, respectively (all p-values <0.01 compared to vehicle treatment, Fig. 1). Similarly, animals dosed with 40 mg/kg TID starting at −1, +24, or +48 h relative to infection had survival rates of 100, 100, and 90%, respectively (all p-values <0.01 compared to vehicle treatment, Fig. 1). Animals dosed with 20 mg/kg TID starting at −1, +24, or +48 h relative to infection had survival rates of 85, 100, and 70%, respectively (all p-values <0.01 compared to vehicle treatment, Fig. 1). Animals dosed with 10 mg/kg TID showed statistically significant survival when dosing started at −1 or +24 h but not +48 h relative to infection, with survival rates of 60 (p < 0.01), 56 (p < 0.05), and 36% (p > 0.05), respectively (Fig. 1). The control groups that were infected with the same lethal ADE DENV challenge but dosed TID with vehicle only (water) had 10–20% survival (Fig. 1). Additionally, the control groups receiving vehicle only lost significantly more weight and had significantly worse health scores as compared to animals dosed with UV-4B (data not shown). Under the conditions of this study, significant increases in survival were observed when mice were dosed at 10 mg/kg TID with treatment delayed for as long as 24 h post-infection; increased survival was observed at the 20 mg/kg TID dose when treatment was delayed as late as 48 h post-infection. In a separate experiment, reduction in viral titers in serum and various tissues was demonstrated in a slightly different ADE DENV2 model (Balsitis et al., 2010, Orozco et al., 2012, Perry et al., 2013, Rathore et al., 2011, Schul et al., 2007, Watanabe et al., 2012) when mice were treated with 100 mg/kg of UV-4 as UV-4B (Supplementary Figure 1).

**Fig. 2:**
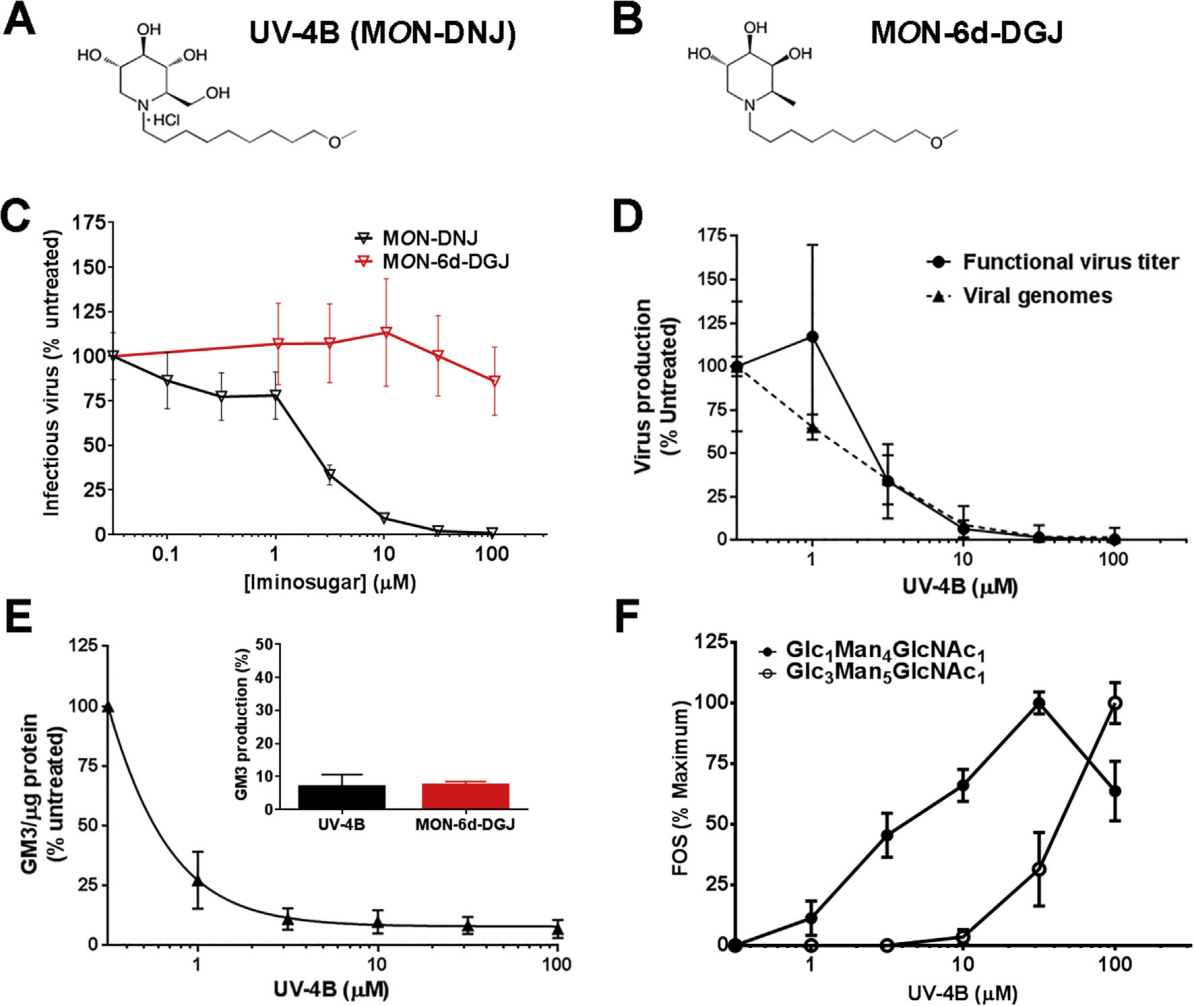
***In vitro* evaluation of iminosugar enzyme inhibition and antiviral activity using human monocyte-derived macrophages (MDMΦs)**. Chemical structure of (A) *N*-(9′-methoxynonyl)-1-deoxynojirimycin hydrochloride (UV-4B, M*O*N-DNJ) and (B) *N*-(9′-methoxynonyl)-1,6-dideoxygalactonojirimycin (M*O*N-6d-DGJ). (C-D) Primary human MDMΦs were infected with DENV2 strain 16681 at a multiplicity of infection of 1 and treated with a titration of iminosugar for 48 h. (C) Infectious virus titer was determined by plaque assay using LLC-MK2 (monkey kidney) cells. Cells from three donors were treated in triplicate and resulting samples quantitated using plaque assays in triplicate on each sample. Counts were normalized to 100% for untreated samples. Data are presented as mean ± SD. (D) Production of functional virus (quantitated using LLC-MK2 plaque assays) conducted on nine technical replicates was compared to total virus secreted (assessed using qRT-PCR conducted in technical duplicate) in untreated cells or cells treated with UV-4B. The values for both RNA and infectious virus are means normalized to untreated samples within a donor and a single representative donor is plotted as mean ± SD. (E) Uninfected human MDMΦs were treated with iminosugars for 48 h and glycolipids were isolated from whole cell lysates. Production of monosialodihexosylganglioside (GM3) was normalized to total protein content for each sample and each treatment was normalized to untreated controls on a donor specific basis. Inhibition of GM3 production by 100 μM of UV-4B (black) or 105 μM of M*O*N-6d-DGJ (red) was assessed (inset) and a range of UV-4B concentrations. Data are presented as mean ± SD from assay of three biological replicates assayed in single sample. (F) MDMΦs were treated in technical duplicate for 48 h with iminosugar with a serial dilution of UV-4B or M*O*N-6d-DGJ (not shown, results were negative) and inhibition of α-glucosidase I was measured by accumulation of Glc_3_Man_5_GlcNAc_1_ (white circles) while inhibition of α-glucosidase II was measured by accumulation of Glc_1_Man_4_GlcNAc_1_ (black circles). For each donor, the maximal concentration of each oligosaccharide species reached was normalized to 100%.

The proposed antiviral mechanism of action of UV-4B is competitive inhibition of ER-resident α-glucosidases I and II. The potential utility of ER α-glucosidases as a pharmacological target capable of conferring broad-spectrum resistance to viral infectivity was demonstrated in a recent publication that characterized two children with a genetic defect resulting in absence of ER α-glucosidase I (Sadat et al., 2014). In spite of significant hypogammaglobulinemia, the children had no history of viral disease, were not able to generate immune responses to live viral vaccines, and their cells did not support replication of phylogenetically divergent viruses including HIV and influenza. To confirm that pharmacological inhibition of the ER α-glucosidases is the mechanism by which UV-4B confers antiviral activity, purified enzyme, cellular, and animal studies using UV-4B with a glucose (M*O*N-DNJ, Fig. 2A) or galactose (*N*-(9′-methoxynonyl)-1,6-dideoxygalactonojirimycin (M*O*N-6-deoxy-DGJ or M*O*N-6d-DGJ, Fig. 2B)) sterochemistry were conducted. Differences in inhibition of glucosidase and glycosidase enzymes specific to sugar stereochemistries (glucose versus galactose) may exist and affect their antiviral activities.

First, human monocyte-derived macrophages (MDMΦs) from a minimum of three donors were infected with DENV2 strain 16681 and then incubated for 48 h in the presence of serial dilutions of UV-4B (M*O*N-DNJ) or M*O*N-6d-DGJ. Infectious virus was quantitated by plaque assay, and only the glucomimetic UV-4B inhibited DENV2 replication (Fig. 2C). The effect on total virus production by UV-4B was then assessed by quantitative reverse transcriptase real-time polymerase chain reaction (qRT-PCR) for DENV NS5 RNA and compared with functional virus load quantitated by plaque assay (Fig. 2D) (Laue et al., 1999, Sayce et al., 2015). Inhibition of infectious (functional) viral titer correlated directly with decrease in total virus secreted (Fig. 2D).

Next, the profile of inhibition of purified glucosidases and glycosidases by UV-4B and M*O*N-6d-DGJ, respectively, was tested as previously described (Asano et al., 1998, Sayce et al., 2015). As shown in Table 2, UV-4B demonstrated inhibition of purified ER α-glucosidases I and II with IC_50_ of 0.16 and 1.8 μM, respectively, while M*O*N-6d-DGJ did not inhibit these enzymes up to the highest concentration tested (1.05 mM). Similar observations were made for both molecules regarding inhibition of other α-glucosidases. Conversely, as expected, only M*O*N-6d-DGJ inhibited α-galactosidase (Table 2). UV-4B appears to be a more potent inhibitor of ceramide glucosyltransferase isolated from the human cell line HL60 than M*O*N-6d-DGJ (IC_50_ of 0.39 and 88.3 μM, respectively). Neither molecule was a strong inhibitor of enzymes responsible for processing β-linkages or non-glucose, non-galactose containing saccharides (data not shown).

Further analyses of the enzymatic activities of the molecules in MDMΦs were undertaken in the absence of DENV infection by detecting glycosphingolipids (GSLs) and free oligosaccharides (FOS) as previously described (Alonzi et al., 2008, Neville et al., 2004). First, MDMΦs were treated for 48 h with 100 μM of UV-4B or 105 μM of M*O*N-6d-DGJ, and whole cell lysates were collected and assayed for monosialodihexosylganglioside (GM3) levels as an indicator for modification of glycolipid processing (Platt et al., 1994a, Platt et al., 1994b). As shown in the inset graph in Fig. 2E, UV-4B and M*O*N-6d-DGJ both reduced GM3 levels (normalized to total protein) by >90%. The dose-dependent relationship of UV-4B on GM3 levels was demonstrated in cells from three donors over a titration of 1–100 μM (Fig. 2E). The lowest dose tested (1 μM) reduced GMS3 levels by an average of 73% and >90% inhibition was observed with 10 μM UV-4B. Generation of FOS is used as a marker of inhibition of ER-resident α-glucosidases and has been previously used in immortalized cell lines and rodents (Alonzi et al., 2008). The generation of FOS was assessed in the MDMΦs after 48 h of treatment with 100 μM of UV-4B or 105 μM of M*O*N-6d-DGJ. FOS that were detected in M*O*N-6d-DGJ treated samples were identified as FOS generated by inhibition of lysosomal β-*N*-acetylhexosaminidases (Glawar et al., 2012), while monoglucosylated or triglucosylated oligosaccharide species, which would be a product of inhibited glycoprotein processing, were not detected (data not shown). In contrast, addition of 100 μM of UV-4B led to accumulation of FOS representative of inhibition of both α-glucosidases, and the full dose-response relationship of treatment with UV-4B and production of FOS is shown in Fig. 2F.

**Fig. 3:**
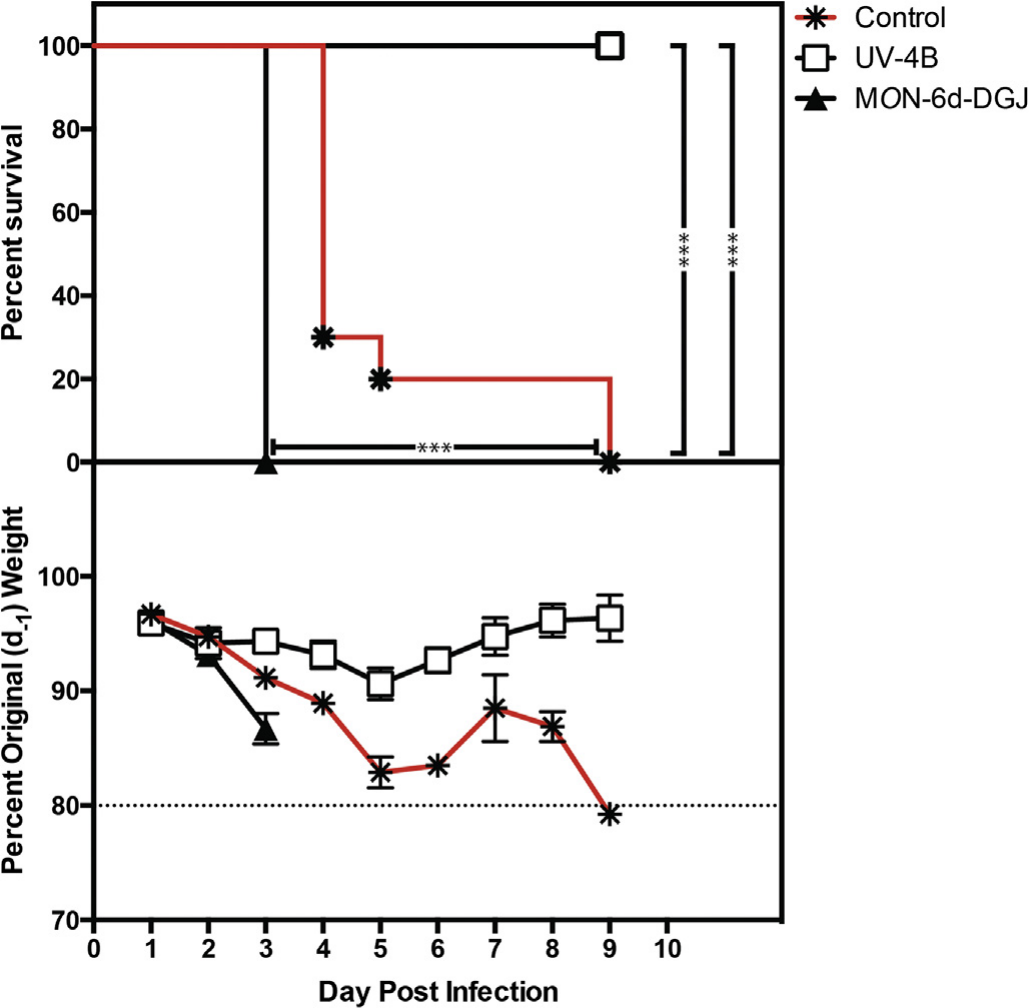
**Antiviral efficacy of UV-4B and M*O*N-6d-DGJ in lethal dengue ADE mouse model**. Groups (n = 10) of male and female AG129 mice, aged 5 weeks, received the first treatment dose of 100 mg/kg of UV-4B and M*O*N-6d-DGJ compound or vehicle (water) orally starting 1 h before infection with DENV2 S221 and administration of DENV-specific antibody clone 2H2 at a dose of ∼1 LD_90_ (10^9^ genomic equivalents); treatment with UV-4B continued every 8 h daily for 7 days. (Top) Survival data are plotted as percent survival against days post-infection. Asterisks denote statistical significance as determined by the Log-rank (Mantel-Cox) test (***P < 0.001). (Bottom) The mean percent weights for each group are plotted compared to their percent weight on Day -1 against days post-infection. Error bars represent the standard error mean (SEM).

Lastly, protection in the DENV ADE mouse model was compared after seven days of treatment with 100 mg/kg TID of UV-4 as UV-4B or the same dose and regimen of M*O*N-6d-DGJ. The results clearly showed antiviral efficacy of UV-4B (p < 0.0001) but not M*O*N-6d-DGJ (Fig. 3). Together, these findings suggest that antiviral activity of UV-4B is mediated by the inhibition of the ER α-glucosidases and not via inhibition of the glycosphingolipid pathway, which is inhibited by both UV-4B and M*O*N-6d-DGJ. These data support and extend the ER α-glucosidases mechanism of action demonstrated for related glucose- and galactose-stereochemistry iminosugars (Sayce et al., 2015).

Our previous studies demonstrated the iminosugar UV-4 and its hydrochloride salt UV-4B are protective against lethal DENV2 challenge and reduce viremia and viral titers in multiple tissues (Perry et al., 2013). Additionally, we demonstrated there is no evidence of escape of DENV via resistance-conferring mutation after multiple passages in mice in the presence of UV-4B (Plummer et al., 2015), and that treatment with UV-4B does not alter the antibody response after DENV infection (Perry et al., 2013). The *in vivo* studies described herein were conducted to further define the minimum effective dose and the therapeutic window for oral dosing of UV-4B in the same ADE model of DENV infection, for the purpose of informing decisions about proposed dosing regimens for future human clinical trials. The effective oral dose of UV-4B given TID for seven days that resulted in statistically significant survival was considered to be 10 mg/kg and 20 mg/kg, when administration was initiated 24 or 48 h after infection, respectively. Additionally, we demonstrated efficacy against a diverse set of DENV1-4 clinical isolates *in vitro*. The UV-4B IC_50_ for the mouse challenge virus S221 is on the higher end and two-fold above the average *in vitro* IC_50_ (∼20uM) of the other 12 isolates (Table 1); therefore, we predict the S221 mouse model is more, rather than less, stringent for assessing the antiviral activity of UV-4B *in vivo*. Further, the studies here confirm that ER α-glucosidase inhibition and not inhibition of glycolipid processing is likely responsible for the antiviral activity of the iminosugar UV-4B. These studies support the clinical development of UV-4B in ongoing Phase 1 (https://clinicaltrials.gov/ct2/show/NCT02061358) and planned Phase 2 clinical trials.

## Figures and Tables

**Table 1:** Summary of antiviral activity based on a virus yield reduction assay of UV-4B against multiple dengue strains in Vero cells. To determine the IC_50_, UV-4B was preincubated for 1 h, the cells were infected with the indicated dengue virus isolates for 1 h at MOI of 0.01 and the media replaced before a five day incubation period. The supernatants were then assessed for functional virus using an immunoplaque assay. Shown are the results for the individual experiments, each as the average of 2–4 replicates along with the calculated average and standard deviation (SD).

		IC_50_ UV-4B [μM]^a^					
Serotype	Isolate	Replicate				Average	SD
		1	2	3	4		
DENV-1	779,172^b^	0.47	3.52	8.52	8.08	5.15	3.85
	SH29177^b^	3.86	0.33	–	–	2.10	2.50
	PRS41393	29.95	45.43	–	–	37.69	10.95
DENV-2	SL 5-17-04^b^	7.42	9.91	41.09	30.96	22.34	16.36
	NGC^c^	5.41	5.84	5.75	8.95	6.49	1.65
	UIS 1288^b^	27.94	12.48	13.46	20.86	18.69	7.21
DENV-3	SL 5-29-04^b^	2.33	2.73	5.38	4.11	3.64	1.39
	UIS 776^b^	8.55	4.58	8.55	4.58	6.56	2.80
	H87	87.60	85.37	–	–	86.49	1.58
DENV-4	779,157^b^	0.90	35.47	–	–	18.18	24.44
	C258/97^b^	9.83	8.06	–	–	8.95	1.25
	H241	2.78	1.42	–	–	2.78	1.42

– Not performed.

a Data shown as IC_50_ are the results for the individual experiments, each as the average of 2–3 replicates within each experiment.

b Clinical isolates were obtained from World Reference Center for Emerging Viruses and Arboviruses at University of Texas Medical Branch.

c Obtained from ATCC (Manassas, Virginia).

**Table 2:** *In vitro* enzyme inhibition by iminosugars.

Class	Enzyme	IC_50_^a^	
		UV-4B	M*O*N-6d-DGJ
α-glucosidase	Mouse ER α-glucosidase I	0.16 μM	>1.05 mM^b^
	Mouse ER α-glucosidase II	1.8 μM	>1.05 mM^b^
	Rat intestinal maltase	0.28 μM	>1.05 mM^b^
	Rat intestinal isomaltase	1.4 μM	>1.05 mM^b^
	Rat intestinal sucrase	0.5 μM	>1.05 mM^b^
	Human lysosome	0.39 μM	>1.05 mM^b^
Glucosyltransferase	HL60^c^	0.39 μM	88.26 μM
α-galactosidase	Human lysosome	>1 mM^b^	255.8 μM

a IC_50_ is the concentration required to inhibit the enzyme to 50% activity.

b The drug did not reach an IC_50_ for the given enzyme up to the maximal dose tested.

c HL60 is a human promyelocytic cell line from which the glucosyltransferase has been isolated.
