# Supplementary material for: Inhibition of endoplasmic reticulum glucosidases is required for in vitro and in vivo dengue antiviral activity by the iminosugar UV-4
Source: Antiviral Res. 2016 May;129:93–8. doi: 10.1016/j.antiviral.2016.03.001 (PMC5064435; doi:10.1016/j.antiviral.2016.03.001)
Supplement: Supplementary file 1 [file mmc1.docx]

**Supplemental Figure 1. UV-4B reduces circulating virus *in vivo*.** AG129 mice were administered 5 μg of 4G2 antibody intraperitoneally 24 hours prior to infection with 10^5^ PFU of D2S10, a mouse-adapted DENV2, to mimic ADE infection. Drug was administered orally immediately following infection and every 8 hours thereafter until mice were sacrificed at 88 hours post-infection (immediately prior to normal time of death in untreated controls). Serum and tissue samples from the UV-4B-treated or untreated mice were collected for all mice 8 hours prior to normal time of death in infected, untreated controls. **(A)** Infectious virus level in the serum samples was assayed by BHK-21 plaque assay. Data are presented as mean ± SD. **(B)** Viremia was reduced with UV-4B treatment as assayed by qRT-PCR. (**C-G**) Tissues were collected and viral load was determined by qRT-PCR. Viral load in genome equivalents (GE) was normalized to GAPDH (ng). The tissues assayed were: (**C**) parenteral lymph nodes, (**D**) small intestine, (**E**) liver, (**F**) kidney, and (**G**) spleen. Individual readings as assessed by qRT-PCR are plotted (triangles), with the median value for each treatment indicated by the horizontal line. Statistically significant differences (*, p<0.05) were assessed by non-parametric Mann-Whitney testing and were observed in the serum, spleen and lymph node samples assessed by qRT-PCR. Data are presented as mean ± SD. Three mice were tested per group and assays were conducted in technical duplicate.
